# Machine learning quantification of hepatobiliary lesion-to-liver contrast enhancement ratio in benign hepatocellular tumors: a feasibility study

**DOI:** 10.1016/j.redii.2026.100066

**Published:** 2026-09-28

**Authors:** Édouard Reizine, Salim Jazzar, Sonia Colombo, Giuseppina Pierro, Silvia Gonella, Deborah Fazzini, Marco Ali, Fabio Tedoldi, Giovanni Valbusa, Frédéric Pigneur, Sébastien Mulé, Alain Luciani

**Affiliations:** a Department of Radiology, APHP, centre hospitalier universitaire Henri-Mondor, Créteil, France; b Faculté de médecine, université Paris Est Créteil, Créteil, France; c Inserm, unité U955, équipe 18, Créteil, France; d Bracco Imaging SpA, Centro Ricerche Bracco, Colleretto Giacosa, TO, Italy; e Centro Diagnostico Italiano, Milan, Italy

**Keywords:** Focal nodular hyperplasia, Adenoma, Liver neoplasms, Organic anion transporters, Contrast media

## Abstract

**Objectives:**

To evaluate the feasibility of a machine learning derived semi-automatic quantification of the lesion-to-liver contrast enhancement ratio following hepatobiliary contrast agent injection for the characterization of benign hepatocellular tumors.

**Materials and methods:**

Precontrast, arterial, portal venous and hepatobiliary phases of liver magnetic resonance images were coregistered, then the liver was automatically segmented by a pretrained U-Net network. Each lesion was then manually segmented on the arterial phase, while different regions of interest were automatically placed in the liver parenchyma (ring shape, circular). Two-dimensional and three-dimensional lesion-to-liver contrast enhancement ratios were automatically calculated using each different regions of interest and the whole liver volume. All the calculated lesion-to-liver contrast enhancement ratio were compared to a manual lesion-to-liver contrast enhancement ratio and correlated to organic-anion-transporting polypeptide expression. Moreover, the ability of each calculated lesion-to-liver contrast enhancement ratio in classifying the two lesions was investigated.

**Results:**

A total of 23 hepatocellular benign tumors were analyzed including nine focal nodular hyperplasias (39 %) and 14 hepatocellular adenomas (61 %). The correlation between each calculated lesion-to-liver contrast enhancement ratio and the manual value was strong (Pearson coefficient ranging between 0.69 and 0.87). Regarding correlation with organic-anion-transporting polypeptide expression, the highest correlation values was obtained with two-dimensional ring region of interest lesion-to-liver contrast enhancement ratio (Pearson coefficient = 0.56). All lesion-to-liver contrast enhancement ratio values were effective in classifying between focal nodular hyperplasia and hepatocellular adenoma, with the best results in terms of inverse Fisher discriminant ratio obtained with ring-shaped region of interest (0.34 2D, 0.28 3D) and uniform liver region of interest (0.28 2D, 0.37 3D).

**Conclusion:**

Semi-automatic quantification of the hepatobiliary lesion-to-liver contrast enhancement ratio both using a two- or a three-dimensional approach is a promising tool for benign hepatocellular characterization.

## Highlights

• All different automatic region of interest were effective for differentiating focal nodular hyperplasia and hepatocellular adenoma.
• Intraclass correlation coefficients were high to very high for all the calculated lesion-to-liver contrast enhancement ratios.
• Results of our study do not support a superiority for the 3D evaluation.

## 1 Introduction

Focal nodular hyperplasia and hepatocellular adenoma are both classified as benign hepatocellular tumors, but their distinction is crucial: the management strategy is mostly conservative for the former, but includes resection, ablation or follow up for the latter [1,2].

Hepatobiliary magnetic resonance imaging (MRI) contrast agents are increasingly being used for liver imaging and has been proven to be helpful to differentiate focal nodular hyperplasia from hepatocellular adenoma [3]. However, visual analysis can be misleading, in particular for lesions with hyperintensity on the T1 precontrast sequence [4]. For those situations, using a quantitative approach of the hepatobiliary uptake seems mandatory. Even if there is no consensus regarding the optimal methods for the quantification, using the lesion-to-liver contrast enhancement ratio showed good results for the differentiation between focal nodular hyperplasia and hepatocellular adenoma, or in subtyping of the latter [4], [5], [6], [7], [8], [9]. Hence, this method also showed good results for the analysis of lesions with indeterminate relative enhancement in visual analysis [4]. Moreover, hepatobiliary MRI contrast agents are characterized by an extracellular distribution combined with a selective uptake by functioning hepatocytes through specific organic-anion polypeptide transporters present on the hepatocytes surface, allowing the acquisition of delayed hepatobiliary images, while prior studies showed that manual lesion-to-liver contrast enhancement ratio was correlated to the molecular background of benign hepatocellular tumor, specifically correlated with the level of transporters expression [7], [9], [10], [11], [12].

Unfortunately, the lesion-to-liver contrast enhancement ratio method suffers from several limitations. Besides the time-consuming manual contouring process, which limits the use of quantitative analysis in clinical routine, the analysis is two-dimensional, and does not allow for an overall enhancement evaluation. Moreover, even if prior studies found good interobserver reproducibility [5,9], a study showed that this method could suffer from external validation [13].

Hence, the purpose of the study was to evaluate the feasibility of a machine learning derived semi-automatic quantification of the lesion-to-liver contrast enhancement ratio following hepatobiliary contrast agent injection for the characterization of benign hepatocellular tumors.

## 2 Methods and materials

### 2.1 Patient population

A dataset of a prior study correlating hepatobiliary lesion to liver relative enhancement after meglumine gadobenate (Gd-BOPTA) injection to organic-anion-transporting polypeptide expression in patients with benign hepatocellular tumors (focal nodular hyperplasia and hepatocellular adenoma) using the lesion-to-liver contrast enhancement ratio was used [7]. Patients were referred at our tertiary referral academic center from 01/09/2009 to 01/03/2013.

A total of 23 hepatocellular benign tumors were analyzed including nine focal nodular hyperplasia (39 %) and 14 hepatocellular adenoma (61 %), corresponding in detail to six of the inflammatory subtype (43 %), two of the HNF1a inactivated subtype (14 %), three of the β-catenin-mutated subtype (21.5 %), and three of the unclassified subtype (21,5 %) according to phenotypic immunohistochemistry findings [14]. The final diagnosis was made on the pathological analysis of resected specimen (*n* = 12) and of targeted liver biopsy (*n* = 11). The mean size of focal nodular hyperplasia and hepatocellular adenoma was not significantly different (27.7 ± 14.8mm vs. 40.9 ± 28.5mm, *p* = 0.38).

### 2.2 MRI technique

All patients underwent MRI (Avanto Fit 1.5T, Siemens or Skyra 3.0T, Siemens). In addition to breath-hold fat-suppressed turbo spin echo T2-weighted images sequences, breath-hold half-Fourier acquisition single-shot turbo spin- echo T2-weighted image sequences and diffusion-weighted images (b = 50 s/mm^2^, b = 400 s/mm^2^, b = 800 s/mm^2^), acquisitions before contrast injection comprised breath-hold in-phase and out-of-phase T1-weighted images, breath-hold three-dimensional (3D) volumetric interpolated breath-hold examination T1-weighted images, fat-suppressed T1-weighted image sequences. After acquisition of unenhanced sequences, meglumine gadobenate contrast medium (Multihance®, Bracco Imaging SpA) was administered at a dose of 0.1 mL/kg (0.05 mmol/kg) body weight as a single intravenous bolus injection followed by a 20-mL saline flush. Conventional dynamic phase images were then acquired. Delayed hepatobiliary acquisitions were acquired after a mean delay of 90 min (range: 70–120 min) following contrast medium injection, using the same IP-OP T1-weighted images, volumetric interpolated breath-hold examination T1-weighted images and T1-weighted image fat-suppressed sequences parameters used for acquisition of pre-contrast sequences. All quantitative analysis were performed only on volumetric interpolated breath-hold examination T1-eighted image sequences.

### 2.3 Manual liver-to-lesion contrast enhancement ratio

Two radiologists in consensus measured the lesion-to-liver contrast enhancement ratio as follows: for each lesion, regions of interest were manually placed on each liver lesion as well as on the adjacent liver parenchyma on the volumetric interpolated breath-hold examination images, before and after contrast medium injection on the hepatobiliary phase images as: 1) Liver lesion regions of interest were manually traced to cover the entire surface of the lesion on the transverse plane that showed the largest diameter of the lesion. The contours of the region of interest precisely matched those of each lesion, even in lesions with irregular borders; 2) A circular region of interest was placed in the adjacent liver parenchyma on the same transverse plane selected for the liver lesion analysis, and in the same anteroposterior position to avoid heterogeneities due to surface coils. The size of these regions of interest was arbitrarily chosen between 100 and 200 mm^2^, taking care to avoid liver lesions or vessels.

For each lesion, the manual lesion-to-liver contrast enhancement ratio defined as m$\text{LLCER}=100.\frac{\left[\left(\frac{\text{SITpost}}{\text{SILVpost}}\right)-\left(\frac{\text{SITpre}}{\text{SILVpre}}\right)\right]}{\frac{\text{SITpre}}{\text{SILVpre}}}$ was calculated, where SITpost and SILVpost are the respective signal intensity of tumor and liver on hepatobiliary phase sequences, and SITpre and SILVpre are the respective signal intensity of tumor and liver before injection on the same pre- and postcontrast volumetric interpolated breath-hold examination sequence.

The mean manual lesion-to-liver contrast enhancement ratio observed in all lesions was determined and considered as reference value.

### 2.4 Preprocessing

Before any preprocessing data were fully anonymized in order to remove any personal data or sensitive information. Volumetric interpolated breath-hold examination T1-weighted image sequences of the selected phases (pre contrast, arterial and hepatobiliary phase) were selected by using a home-developed plugin working on Slicer (a free, open-source software for visualization, processing, segmentation, registration, and analysis of medical images). The plugin allows to visualize all the acquired scans of a certain patient and to select those that were considered relevant for the study. After selection the plugin saves the images introducing a conversion form dicom format to .nrrd. The preprocessing pipeline used in this work did not include any resampling nor rescaling and consisted of two following steps: N4 bias field correction algorithm (applied to all phases for correcting low frequency intensity non-uniformity present in MRI image data) and spatial registration (to guarantee a precise overlapping of liver and liver lesions among the different T1 weighted pre- and post-contrast images).

N4 bias field correction was performed using the SimpleITK class N4BiasFieldCorrectionImageFilter. The algorithm is a variation of the original N3 (Nonparametric nonuniform intensity normalization) introduced in 1998 as a method for correcting nonuniformity associated with magnetic resonance images. The method assumes a simple parametric model (Gaussian) for the bias field and does not require tissue class segmentation. N4 algorithm has the additional benefits of an improved B-spline fitting routine which allows for multiple resolutions to be used during the correction process. [15]

The coregistration strategy adopted consisted in a pairwise method using mutual information as metric applied to all sequences in cascade (i.e., arterial coregistered on precontrast; portal venous on arterial; hepatobiliary on venous). Parameters of the transformation were set according to map No. 47 of Model Zoo Elastix database [15] specifically designed and published for four-dimensional (4D) dynamic contrast enhanced abdominal MRI [16]. All coregistered sequences were visually checked to assess correct spatial alignment and motion correction.

In the case of failure of the coregistration step, the whole workflow was applied to original sequences.

Then all the lesions were manually segmented in 3D by the two radiologists.

### 2.5 Automatic lesion-to-liver contrast enhancement ratio

In order to allow automatic region of interest positioning inside the healthy liver parenchyma, segmentation of the whole hepatic tissue is required. Liver was automatically segmented separately on precontrast and hepatobiliary phase by a nn-U-Net network [17] pretrained on an external dataset (45 patients retrospectively collected during the clinical study approved by Comitato Etico Milano Area 2 number 452_2022bis and manually annotated by two post graduate medical students by means of Slicer). nn-U-Net is a self-configuring method for deep learning-based biomedical image segmentation that automatically adapts preprocessing, network architecture, training and postprocessing for the specific segmentation task, thus improving performances of traditional U-Net architectures.

Preprocessing step was adapted to the fingerprint of our specific dataset, and included a resampling step at spacing 2 × 2 × 2 mm and Z-score normalization (during training and inference, each image is normalized independently by first subtracting its mean and then dividing by its standard deviation).

This training ran on Pythorch and was configured as a 2D U-Net operating on axial slices (corresponding to the two axes with the highest resolution).

Based on the numerosity of our starting dataset, a batch size of 90 was selected by nn-U-Net autoconfiguration. A fixed training scheme starting form randomly initialized weights was then applied to the 2D nn-U-Net configuration. The network was trained for 1000 epochs, with one epoch being defined as iteration over 250 mini-batches. Stochastic gradient descent with Nesterov momentum (µ = 0.99) and an initial learning rate of 0.01 is used for learning network weights. For each deep supervision output, a corresponding downsampled ground truth segmentation mask is used for loss computation. The training objective is the sum of the losses (L) at all resolutions, L = w1 × L1 + w2 × L2 +… H, where L is a composite loss resulting from the sum of cross-entropy and Dice loss.

A variety of data augmentation techniques have been applied on the fly during training: rotations, scaling, Gaussian noise, Gaussian blur, brightness, contrast, simulation of low resolution, gamma correction and mirroring. [17]

A total of five folds were trained on the dataset. Ensembling was implemented, so that the final prediction was obtained by averaging the softmax predictions of the five models from the folds.

All predictions are resampled back to the original spacing of the MRI prior to the following steps.

Automatic postprocessing was applied to segmentations to only preserve major interconnected components. When automatic segmentation was not adequate for region of interest positioning, an expert radiologist applied manually corrections.

Automatic regions of interest drawing was implemented by a python script embedded within the software 3D Slicer. The integrated application allowed to load both the whole set of volumetric interpolated breath-hold examination images acquired at different vascular phases and the automatic liver segmentation for each patient. After loading volumes and liver mask, two radiologists (independently and blinded to the pathological diagnosis and to the manual lesion-to-liver contrast enhancement ratio) performed a 3D manual segmentation of the lesion(s) on the precontrast and on the hepatobiliary phases. Finally, the following regions of interest were automatically placed in the liver parenchyma:

• Circle region of interest: a circular area (4 pixel radius) was placed in the slice corresponding to the major axial section of the lesion trough the following multi-step procedure: 1) the liver mask of the selected slice is modified excluding 1 cm perimetral region of liver and lesion (Fig. 1, panel a); 2) a min-max normalization is applied to the remaining area; 3) Felzenswalb segmentation is applied in order to identify different signal distribution populations within such region (i.e. liver parenchyma, vessel, signal heterogeneities, etc.; in Fig. 1, panel b each colored region represents a different signal distribution); 4) the predominant cluster is selected, under the assumption that the largest population represents the uniform area of liver parenchyma; 5) an iterative algorithm places a circular regions of interest (Fig. 1, panel c) preferentially at the same anterior-posterior position of the lesion, or in the closest position, with at least 50 % of the pixel within the liver parenchyma cluster is then applied. The algorithm starts placing the center of the region of interest within the liver parenchyma cluster at the horizontal coordinate closest to the lesion. If at least 50 % of the pixel of the circular region of interest fall within the predominant cluster the region of interest is accepted, otherwise a new center location is sought for. The algorithm scan firstly the horizontal coordinates, if no acceptable region is found, the center of the region of interest is moved along the vertical coordinate, selecting first the horizontal coordinates that minimize the distance with respect to the lesion and verifying if the new position is acceptable. The process is iterated until the acceptance criterion is satisfied. In Fig. 1 a schematic representation of the procedure is shown.

**Fig. 1:**
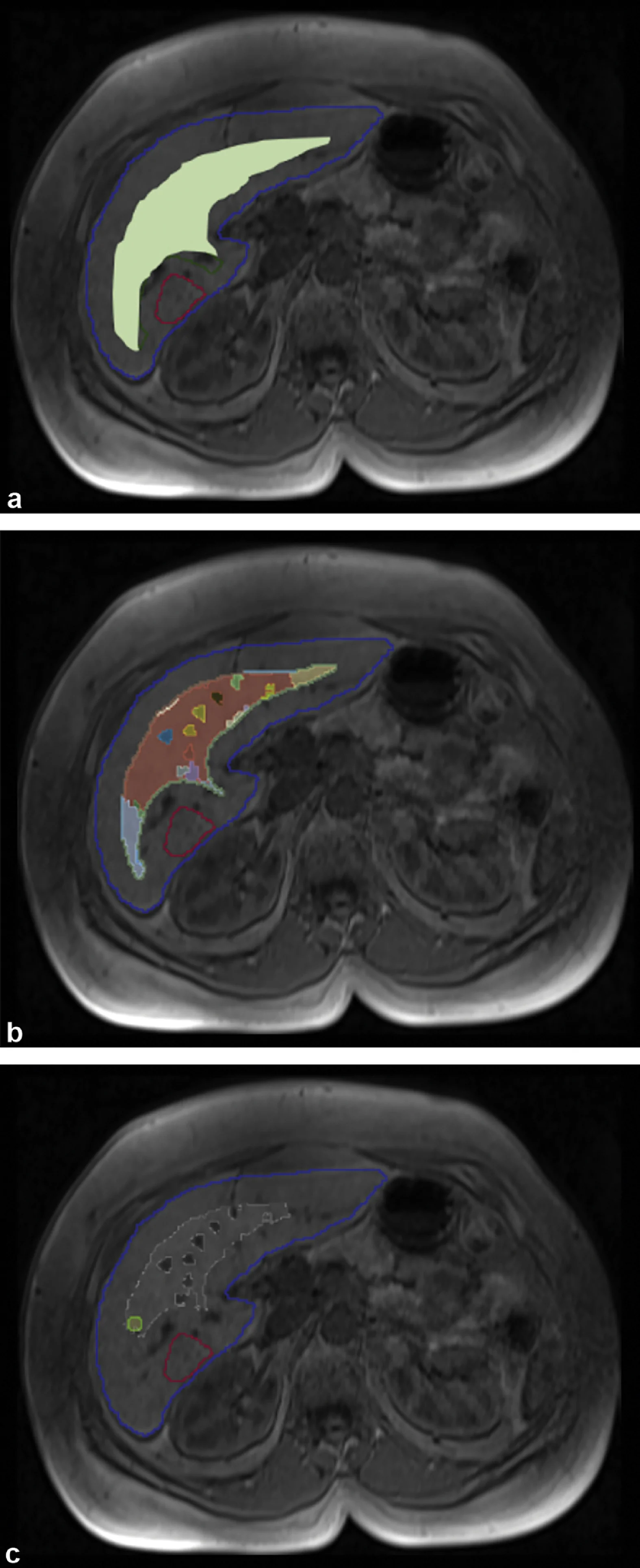
Feasibility study on machine learning derived semi-automatic quantification of the lesion-to-liver contrast enhancement ratio: schematic representation of region of interest circle positioning process. Panel a: original liver mask (blue line) is modified excluding 1 cm perimetral region from liver border and lesion (red line) leading to a reduced mask (light green region). Panel b : results of Felzenswalb segmentation, each colored region corresponds to a different signal distribution, the largest one being homogeneous liver parenchyma. Panel c: circle region of interest positioning within the predominant cluster region (light gray line).
• Ring region of interest: a 1 cm ring-shaped area was drawn 1 cm from the lesion and liver boundaries. For 2D computation only the slice corresponding to the largest transverse section of the lesion was considered, while for 3D computation the ring region of interest was drawn on all slices containing the lesion.
• Whole liver region of interest: a mask including the liver segmentation with exclusion of the lesion was created. For 2D computation only the slice corresponding to the largest transverse section of the lesion was considered, while for 3D computation, 3D liver and lesion segmentation were considered.

A representative example of 2D regions of interest positioning is presented in Fig. 2.

**Fig. 2:**
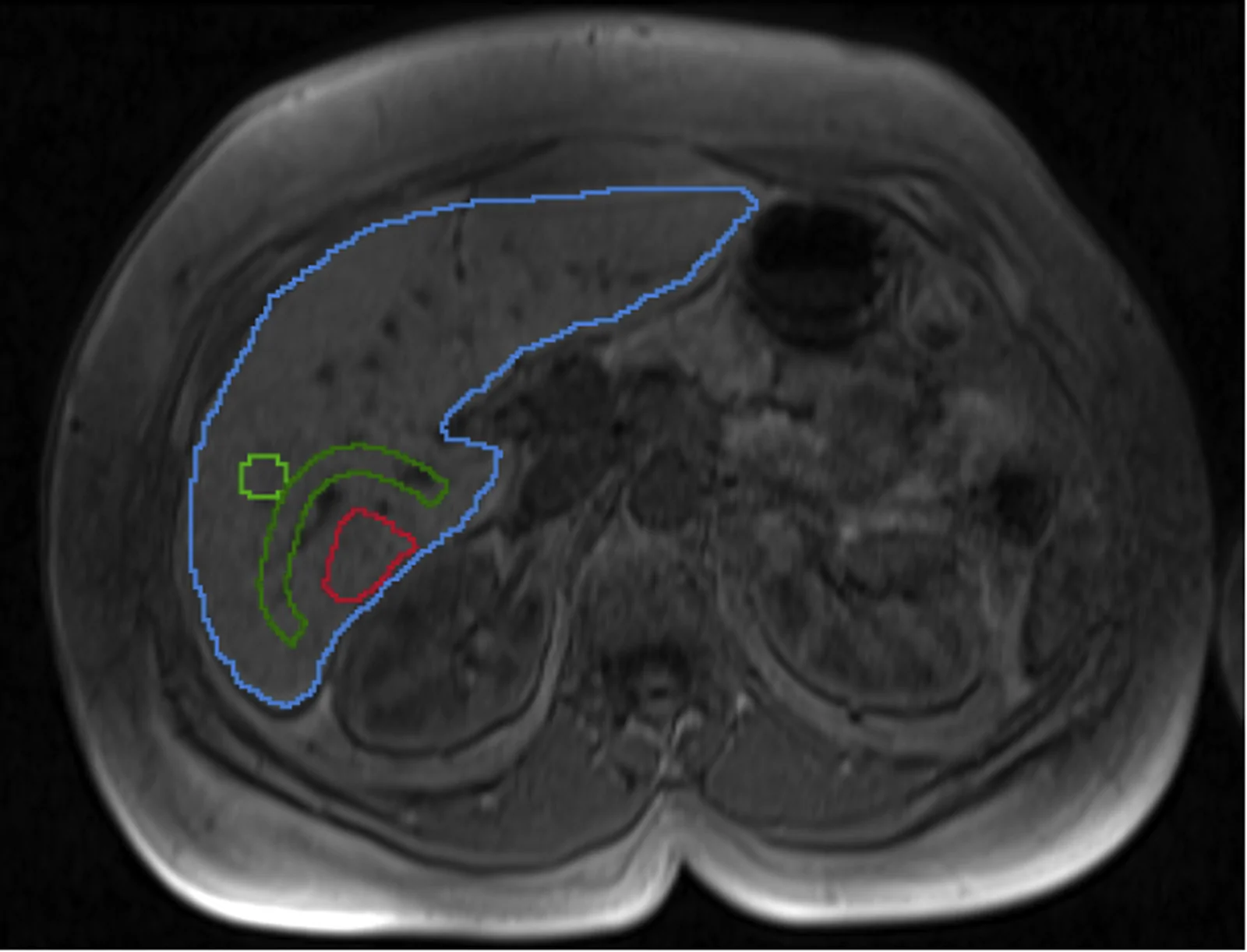
Feasibility study on machine learning derived semi-automatic quantification of the lesion-to-liver contrast enhancement ratio: different regions of interest automatically placed in liver parenchyma in bidimensional axial view. Lesion (red), circle region of interest (light green), ring region of interest (dark green), uniform liver region of interest (blue).

For each automatic region of interest positioning (three shapes, each of one 2D or 3D), the corresponding calculated lesion-to-liver contrast enhancement ratio value was computed, leading to six different estimators for each reader (2D circle, 2D ring, 2D liver, 3D circle, 3D ring, 3D liver).

### 2.6 Statistical analysis

Interobserver reliability between the two readers was measured by two-way mixed model intraclass correlation coefficient for each calculated lesion-to-liver contrast enhancement ratio. Correlation between manual ration and each calculated lesion-to-liver contrast enhancement ratio was estimated by Pearson correlation coefficient for each reader. Correlation between lesion-to-liver contrast enhancement ratio values and organic-anion transporter expression was also computed using Spearman correlation coefficients. Moreover, the ability of each calculated lesion-to-liver contrast enhancement ratio in classifying each lesion as focal nodular hyperplasia or hepatocellular adenoma was investigated using the inverse Fisher discriminant ratio [18].

## 3 Results

### 3.1 Interobserver reliability

Intraclass correlation coefficients for all calculated lesion-to-liver contrast enhancement ratios are presented in Table 1. Results from the two readers showed a very high correlation, with the highest values for 3D ring and for 3D whole liver lesion-to-liver contrast enhancement ratio.

**Table 1:** Results of a feasibility study on machine learning derived semi-automatic quantification of the lesion-to-liver contrast enhancement ratio: intraclass correlation coefficients for all calculated ratios.

	Intraclass correlation coefficient
2D circle lesion-to-liver contrast enhancement ratio	0.77
3D circle lesion-to-liver contrast enhancement ratio	0.84
2D ring lesion-to-liver contrast enhancement ratio	0.88
3D ring lesion-to-liver contrast enhancement ratio	0.97
2D whole liver lesion-to-liver contrast enhancement ratio	0.93
3D whole liver lesion-to-liver contrast enhancement ratio	0.98

2D: two-dimensional; 3D: three-dimensional.

### 3.2 Correlation between calculated, manual lesion-to-liver contrast enhancement ratio and organic-anion transporter expression

The correlation between each calculated lesion-to-liver contrast enhancement ratio and the reference manual ratio is presented in Table 2 for both readers. The correlation between each calculated lesion-to-liver contrast enhancement ratio and the manual ratio value was strong. The highest correlation values were obtained with 2D ring and 3D ring region of interest lesion-to-liver contrast enhancement ratio for both readers.

**Table 2:** Results of a feasibility study on machine learning derived semi-automatic quantification of the lesion-to-liver contrast enhancement ratios: correlation values of calculated ratios with manual ratios (Pearson coefficients).

	Reader 0	Reader 1
2D circle lesion-to-liver contrast enhancement ratio	0.73	0.69
3D circle lesion-to-liver contrast enhancement ratio	0.73	0.69
2D ring lesion-to-liver contrast enhancement ratio	0.83	0.87
3D ring lesion-to-liver contrast enhancement ratio	0.82	0.86
2D whole liver lesion-to-liver contrast enhancement ratio	0.75	0.79
3D whole liver lesion-to-liver contrast enhancement ratio	0.72	0.76

2D: two-dimensional; 3D: three-dimensional.

The correlation values between lesion-to-liver contrast enhancement ratio and organic-anion transporter expression in the whole cohort and in the subgroup of patients with resected specimens are shown in Table 3.

**Table 3:** Results of a feasibility study on machine learning derived semi-automatic quantification of the lesion-to-liver contrast enhancement ratio: correlation values of liver-to-lesion contrast enhancement ratios with organic-anion-transporting polypeptide expression (Pearson coefficients).

	Whole cohort		Subgroup of resected specimens	
	Reader 0	Reader 1	Reader 0	Reader 1
Manual lesion-to-liver contrast enhancement ratio	0.67		0.93	
2D circle lesion-to-liver contrast enhancement ratio	0.35	0.30	0.65	0.34
3D circle lesion-to-liver contrast enhancement ratio	0.38	0.28	0.64	0.33
2D ring lesion-to-liver contrast enhancement ratio	0.51	0.54	0.78	0.76
3D ring lesion-to-liver contrast enhancement ratio	0.51	0.56	0.72	0.77
2D whole liver lesion-to-liver contrast enhancement ratio	0.33	0.39	0.51	0.62
3D whole liver lesion-to-liver contrast enhancement ratio	0.32	0.31	0.37	0.38

2D: two-dimensional; 3D: three-dimensional.

Besides with that of manual lesion-to-liver contrast enhancement ratio, the highest correlation with organic-anion-transporting polypeptide was obtained with 2D ring region of interest ratio.

### 3.3 Differentiation of hepatocellular adenoma and focal nodular hyperplasia

A graphical representation of all lesion-to-liver contrast enhancement ratio values comparing focal nodular hyperplasia and hepatocellular adenoma is shown in Fig. 3 for both readers. For both readers, all calculated lesion-to-liver contrast enhancement ratio values were significantly different between focal nodular hyperplasia and hepatocellular adenoma as shown in Table 4.

**Fig. 3:**
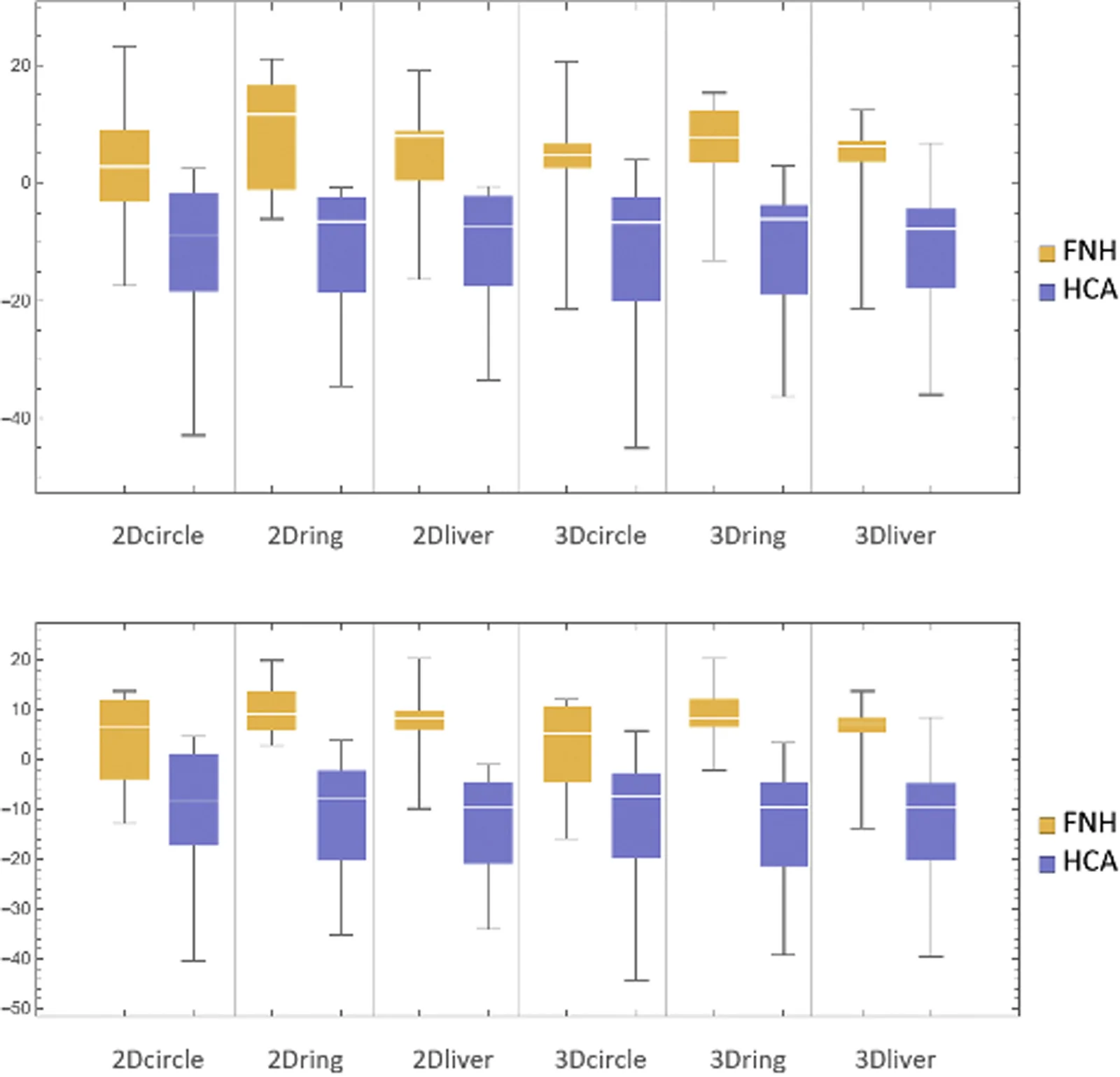
Feasibility study on machine learning derived semi-automatic quantification of the lesion-to-liver contrast enhancement ratio: boxplots of the different calculated lesion-to-liver contrast enhancement ratio in both reader comparing focal nodular hyperplasia (FNH) and hepatocellular adenoma (HCA).

**Table 4:** Results of a feasibility study on machine learning derived semi-automatic quantification of the lesion-to-liver contrast enhancement ratio: comparison of calculated ratios between focal nodular hyperplasia and hepatocellular adenoma.

	Reader 0			Reader 1		
	Focal nodula hyperplasia	Hepatocellular adenoma	*p*	Focal nodula hyperplasia	Hepatocellular adenoma	*p*
2D circle lesion-to-liver contrast enhancement ratio	−9 (−18, −2)	3 (−2, 8)	0.007	−8 (−17, 0)	6 (−4, 12)	0.009
3D circle lesion-to-liver contrast enhancement ratio	−7 (−19, −2)	5 (3, 6)	< 0.001	−7 (−18, −3)	5 (−4, 10)	0.009
2D ring lesion-to-liver contrast enhancement ratio	−7 (−17, −3)	12 (0, 17)	< 0.001	−8 (−18, −3)	9 (6, 13)	< 0.001
3D ring lesion-to-liver contrast enhancement ratio	−6 (−17, −4)	8 (4, 12)	< 0.001	−10 (−19, −5)	8 (7, 12)	< 0.001
2D whole liver lesion-to-liver contrast enhancement ratio	−7 (−18, −3)	8 (3, 9)	0.002	−9 (−21, −5)	8 (6, 9)	< 0.001
3D whole liver lesion-to-liver contrast enhancement ratio	−8 (−18, −4)	6 (4, 7)	0.002	−9 (−20, −5)	7 (6, 8)	0.002

2D: two-dimension; 3D: three-dimension.

All lesion-to-liver contrast enhancement ratio values were also effective in correctly classifying focal nodula hyperplasia and adenoma, with the best results in terms of inverse Fisher discriminant ration as presented in Table 5, obtained with manual lesion-to-liver contrast enhancement ratio (0.35), ring-shaped region of interest (0.34 2D, 0.28 3D), uniform liver region of interest (0.28 2D, 0.37 3D) and lastly circular region of interest (0.40 2D, 0.39 3D).

**Table 5:** Results of a feasibility study on machine learning derived semi-automatic quantification of the liver-to-lesion contrast enhancement ratio: inverse Fisher discriminant ratio for different liver-to-lesion contrast enhancement ratio values and for each reader.

	Inverse Fisher discriminant ratio	
	Reader 0	Reader 1
Organic-anion transporter expression	2.75	
Manual lesion-to-liver contrast enhancement ratio	0.35	
2D circle lesion-to-liver contrast enhancement ratio	1.26	1.17
3D circle lesion-to-liver contrast enhancement ratio	1.24	1.34
2D ring lesion-to-liver contrast enhancement ratio	0.56	0.37
3D ring lesion-to-liver contrast enhancement ratio	0.66	0.47
2D whole liver lesion-to-liver contrast enhancement ratio	0.89	0.43
3D whole liver lesion-to-liver contrast enhancement ratio	1.05	0.64

2D: two-dimensional; 3D: three-dimensional.

## 4 Discussion

The purpose of the study was to evaluate the feasibility of a semi-automatic quantification of hepatobiliary lesion-to-liver contrast enhancement ratio for the characterization of benign hepatocellular tumors. Using a previously published dataset of 23 benign hepatocellular tumors, results of this study suggests that a machine learning approach is a promising tool to quantitatively differentiate hepatocellular adenoma and focal nodular hyperplasia.

For several years, we have known that visual analysis alone of the hepatobiliary phase signal can be misleading, as a significant number of hepatocellular adenomas may appear hyperintense on hepatobiliary phase imaging [4] . This finding is particularly relevant in the setting of underlying hepatic steatosis [4]. Therefore, quantitative enhancement analysis allows a better distinction between lesions showing enhancement and those that do not. In this context, the distinction is not simply binary between focal nodular hyperplasia and hepatocellular adenoma; rather, it helps reinforce the suspicion of focal nodular hyperplasia based on the other imaging sequences or, in lesions not suggestive of focal nodular hyperplasia, may raise the possibility of certain adenoma subtypes or even hepatocellular carcinoma [19].

First, comparing segmentations of two radiologists, intraclass correlation coefficients were high to very high for all the calculated lesion-to-liver contrast enhancement ratio ranging from 0.77 to 0.97. Yet, those values are even higher than what was reported in the first paper evaluating the lesion-to-liver contrast enhancement ratio [6]. This is a major result as, even if several studies showed the clinical interest of calculating the lesion-to-liver contrast enhancement ratio to differentiate focal nodular hyperplasia and hepatocellular adenoma [6,7] or even in the adenoma subtyping [8,9], one study failed to reproduce similar results in an external evaluation [13]. Hence, using a machine learning approach and a semi-automatic quantification could avoid all the biases related to the manual evaluation.

Second, this study allowed to evaluate and compare different automatic placement of region of interest in the liver parenchyma to calculate the lesion-to-liver contrast enhancement ratio and all were effective for differentiating focal nodular hyperplasia and hepatocellular adenoma. The circle region of interest was evaluated to copy what is performed for the manual lesion-to-liver contrast enhancement ratio. However, the placement of this region of interest could be more impacted by heterogeneities due to surface coils; in our study results of the lesion-to-liver contrast enhancement ratio based on the circle region of interest were overall less accurate. The ring region of interest was placed around the lesion at 1 cm from the lesion boundaries to avoid the interface lesion to liver. This method better reflects what is usually performed by the pathologist where the liver parenchyma assessed is closed to the lesion. The last method included the whole liver segmentation which could be particularly useful for patients with heterogeneous liver enhancement on the hepatobiliary phase. Moreover, this method could be helpful to automatically assess the liver function on the hepatobiliary phase.

Finally, all these methods were evaluated either in two and three dimensions. Results of our study do not support a superiority for the three-dimensional evaluation. This is important, as two-dimensional evaluation could be easier to achieve in an automatic lesion segmentation task goal.

Besides the retrospective design this study had several limitations. First, the number of included lesions is low. This specific dataset was chosen given the precise phenotype made by the pathologists particularly the quantitative organic-anion transporter evaluation which provide an accurate reference standard for the hepatobiliary contrast enhancement. Second all lesions segmentations were manually conducted in the study. However, the purpose of this study was to evaluate the feasibly of this approach comparing different segmentations. A study including a larger number of lesion and developing a semi-automatic segmentation of the lesion can now be performed based on this study results. Furthermore, a recent study demonstrated performance similar to lesion-to-liver contrast enhancement ratio using Gd-EOB-DTPA [20], yet a future study evaluating this semi-automated method with another contrast agent should be conducted.

## 5 Conclusion

Semi-automatic quantification of the hepatobiliary lesion to liver contrast enhancement ratio both using a two- or three-dimensional approach is a promising tool for benign hepatocellular lesion characterization, with 2D uniform liver and 3D ring around the lesion being the most accurate region of interest to calculate rapidly and repeatably the lesion-to-liver contrast enhancement ratio.

## Statement for studies in humans

Patients were referred at our tertiary referral academic center from 01/09/2009 to 01/03/2013.

This retrospective study was approved by the local institutional review board the 31/10/2013 (no. IRB 00003835) and informed consent was waived.

## Funding

This work was supported with a financial support by Bracco Imaging, IT.

## Guarantor

The scientific guarantor of this publication is Pr. Alain Luciani.

## Statistics and biometry

One of the authors (SM) has significant statistical expertise.

## Informed consent

Written informed consent was waived by the institutional review board.

## Methodology

Retrospective.

Diagnostic study.

Monocentruc study.

## Declaration of competing interest

Five authors are Bracco Imaging employees. The remaining authors, who are not employees of or consultants for Bracco imaging, had control of inclusion of all data and information that might present a conflict of interest for authors who are employees of Bracco Imaging.
